# Supplementary material for: Subinhibitory antibiotic concentrations promote the excision of a genomic island carried by the globally spread carbapenem-resistant Klebsiella pneumoniae sequence type 258
Source: Microb Genom. 2023 Dec 11;9(12):001138. doi: 10.1099/mgen.0.001138 (PMC10763509; doi:10.1099/mgen.0.001138)
Supplement: Supplementary material 1 [file mgen-9-1138-s001.pdf]

Supplemental Material

Subinhibitory antibiotic concentrations promote the excision of a genomic island carried by the globally spread carbapenem-resistant *Klebsiella pneumoniae* ST258

Alejandro Piña-Iturbe[1], Guillermo Hoppe-Elsholz[1], Isidora D. Suazo[1], Alexis M. Kalergis[1,2], Susan M. Bueno[1#]

[1]Millennium Institute on Immunology and Immunotherapy, Facultad de Ciencias Biológicas, Pontificia Universidad Católica de Chile, Santiago, Chile, 8331150.

[2]Departamento de Endocrinología, Facultad de Medicina, Pontificia Universidad Católica de Chile, Santiago, Chile, 8330023.

[#]Correspondence: Susan M. Bueno (sbueno@bio.puc.cl)

Nucleotide sequences determined by Sanger capillary sequencing. The repeated sequences are underlined.

```
>attB_KP35 Fw primer
TTATCTGACACATTTCAATGATGATTGAGAAATGGATATCCTGCAAAGTATTTTCCGTAATAATTGTCGGAAA
TGACAACAAATTGTCGATGTAAGCGGCAGTTGCACCTGAACAGGCGAATTGATGTTTGACAAAAGTTTTTTCG
CCGCTAAGATACGACTCCACACGATTCCTCTGTAGTTCAGTCGGTAGAACGGCGGACTGTTAATCCGTATGTC
ACTGGTTCGAGTCCAGTCAGAGGAGCCAAATTTAAGAAGCCTGCTTTCGAGCAGGCTTTTTGCTTTTTTGCGT
CGCTGACGTTAACTCAGCATCGCATCCAGCCTGGCAAGCACCTCGTTGACAACGACGTCAGGTATGCGTTCCA
GACGCTTGCCTTTCCCTACCGCCTCCCTACGCCCCGCGCGTCCTCCGTGAACGCACCCCAGCTCAGCCTGAAA
AAGCGCCTGCCAGTAGGATGAATTCACTACCGTTTGGGGGGTGAGATTA
```

```
>attB_KP35 Rev primer
CACTCGACGCCGTACCCGACTCGGCGGCGCTTTTTAGGCTGAGCTGGGGTGCGTTCACGGAGGACGCGCGGGG
CGTAGGGAGGCGGTAGGGAAAGGCAAGCGTCTGGAACGCATACCTGACGTCGTTGTCAACGAGGTGCTTGCCA
GGCTGGATGCGATGCTGAGTTAACGTCAGCGACGCAAAAAAGCAAAAAGCCTGCTCGAAAGCAGGCTTCTTAA
ATTTGGCTCCTCTGACTGGACTCGAACCAGTGACATACGGATTAACAGTCCGCCGTTCTACCGACTGAACTAC
AGAGGAATCGTGTGGAGTCGTATCTTAGCGGCGAAAAAACTTTTGTCAAACATCAATTCGCCTGTTCAGGTGC
AACTGCCGCTTACATCGACAATTTGTTGTCATTTCCGACAATTATTACGGAAAATACTTTGCAGGATATCCAT
TTCTCAATCATCATTTGAAATGTTGTTTCAACATTTCTCACTAAGGTCTCTCTTGACAGTTCACCTCCAAA
```

```
>attP_KP35 Fw primer
CACAGTGAGAGCAGATAGTGATCTTGGATGTAAGTGCTTTAATCTTGACTAAACATCGTTTCTTTACTAAAAA
TTAACTAATTGTTTTATATCAATTACTTTAACAAAAATAACGACAACATCATGTTGATAGATAAAGTTAAATA
AGATTCGTATAGTTAGATGGTTATGACAACAACCAATTGTTTTTAATTGACATTTTATTGATAAGAAAACAAT
AATGATGGTAAATATGTTGGTAAAAATATAGTTAAACTTAACAAAATCAGTAACAACAGGAGATACAGATGAA
GTTCGAGTCCAGTCAGAGGAGCCAATTCAAGGGAAGCAGACGTTCACTGACGTCTGCTTTCTGCTTTTCTATC
AATTGGTTATCCCTCTTCAGTAGTTCACCCTCGTTCACTAAAAACCACTCGAAGCCATATCATTTTGATGGTA
AAAATGCTGGTAATGCTGGTTCGATTTCCCTTTTACCAACAAACGAGGGGATATTTTCATGTCACTGACTGAT
ATCAAAGCAAAAAATGCAAAACCCCTTGAGAAGGAATACAAGCTGACTGATGGCTTTGGTATGTTCCTTCGCG
TTACCCCGAAGGGTTCCAAATACTGGCAAATGGCTTACCGTTCCAAAGGGGAAAAAATAATG
```

```
>attP_KP35 Rev primer
CATTGGTACGTTCGGGGTACGCGAGGACATACCAAAGCCATCAGTCAGCTTGTATTCCTTCTCAAGGGGTTTT
GCATTTTTTGCTTTGATATCAGTCAGTGACATGAAAATATCCCCTCGTTTGTTGGTAAAAGGGAAATCGAACC
AGCATTACCAGCATTTTTACCATCAAAATGATATGGCTTCGAGTGGTTTTTAGTGAACGAGGGTGAACTACTG
AAGAGGGATAACCAATTGATAGAAAAGCAGAAAGCAGACGTCAGTGAACGTCTGCTTCCCTTGAATTGGCTCC
TCTGACTGGACTCGAACTTCATCTGTATCTCCTGTTGTTACTGATTTTGTTAAGTTTAACTATATTTTTACCA
ACATATTTACCATCATTATTGTTTTCTTATCAATAAAATGTCAATTAAAAACAATTGGTTGTTGTCATAACCA
TCTAACTATACGAATCTTATTTAACTTTATCTATCAACATGATGTTGTCGTTATTTTTGTTAAAGTAATTGAT
ATAAAACAATTAGTTAATTTTTAGTAAAGAAACGATGTTTAGTCAAGATTAAAGCACTTACATCCAAGATCAC
TTATCTGCTCTCACACCATTTTGAAAACCCGCCCCTCTTTCCCACTTTTCAAAACAG
```

>725bp_product Fw primer

CGGGACTGGTGGGACATTATGCAGTTGTCGGGGTTTGTTTCCGGGCAAAAAATACAGCCTGAAGGG
GTCAGCCATACCATTCTGCTGATCCTCAGCGTCGGCACTCTGGTGGTGTTAGCCTGCGGCTTCCTC
GTCTCGCTGCGCTTCAAGCTCAACCTGCACACCCACAGCGTCCTGCGCAGCGAGACCCTCAGAATG
CGCGAGCTCGGCTACGCTCAGTCGGAACAGACCAGTGCGGAACACCGCGCGGTGGTCGAGCTGCTG
GCGGGGATGCCTTACGACTGTCTGTGGGGCAATAACAATATTGGCTATCTGAATCGTCATAAACCT
GCTGCCCCGGCGCTCATTAAGGGCGGCGCTTTGAATTCGACATATACCCGAGGTTAACAACATGAA
AGTTTGGCCGGTCAAACATAGCCCATTACTGCGTCAGCCTGAGCGTTTTATCGCCCGCAGCGAACT
GCAGGCGCTGATCCGCAACGTGACGCAAAACCTGGTGAATATTAAGGATGAGAGCGGGCAATTTTT
ACTACGCCTGGATGACGGGCGCGTGATCGATACCAAAGGCTGGGCCGGCTGGGAGTGGACCCACGG
CGTTGGGCTGTACGGCATCTATCAGTATTACCAGCAAACCGGCGATATCGAGATGCGTGATATCAT
CGATCGCTGGTTTGCCGACCGCTTCGCCGAGGGAGCAACGACCAAAAACGTCAATACTATGGCCCC
GTTTCTGACCCTGGCTTACCGTTTGGAAAAGGGAAA

>725bp_product Rev primer

GAGGGCATAGTATTGACGTTTTTGGTCGTTGCTCCCTCGGCGAAGCGGTCGGCAAACCAGCGATCG
ATGATATCACGCATCTCGATATCGCCGGTTTGCTGGTAATACTGATAGATGCCGTACAGCCCAACG
CCGTGGGTCCACTCCCAGCCGGCCCAGCCTTTGGTATCGATCACGCGCCCGTCATCCAGGCGTAGT
AAAAATTGCCCGCTCTCATCCTTAATATTCACCAGGTTTTGCGTCACGTTGCGGATCAGCGCCTGC
AGTTCGCTGCGGGCGATAAAACGCTCAGGCTGACGCAGTAATGGGCTATGTTTGACCGGCCAAACT
TTCATGTTGTTAACCTCGGGTATATGTCGAATTCAAAGCGCCGCCCTTAATGAGCGCCGGGGCAGC
AGGTTTATGACGATTCAGATAGCCAATATTGTTATTGCCCCACAGACAGTCGTAAGGCATCCCCGC
CAGCAGCTCGACCACCGCGCGGTGTTCCGCACTGGTCTGTTCCGACTGAGCGTAGCCGAGCTCGCG
CATTCTGAGGGTCTCGCTGCGCAGGACGCTGTGGGTGTGCAGGTTGAGCTTGAAGCGCAGCGAGAC
GAGGAAGCCGCAGGCTAACACCACCAGAGTGCCGACGCTGAGGATCAGCAGAATGGTATGGCTGAC
CCCTTCAGGCTGTATTTTTTGCCCGGAAACAAACCCCGACAACTGCATAATGATCCCCACCAGCAT
CACCGCCCCCGCCCCTCTTTGGCCACATTTACAAA

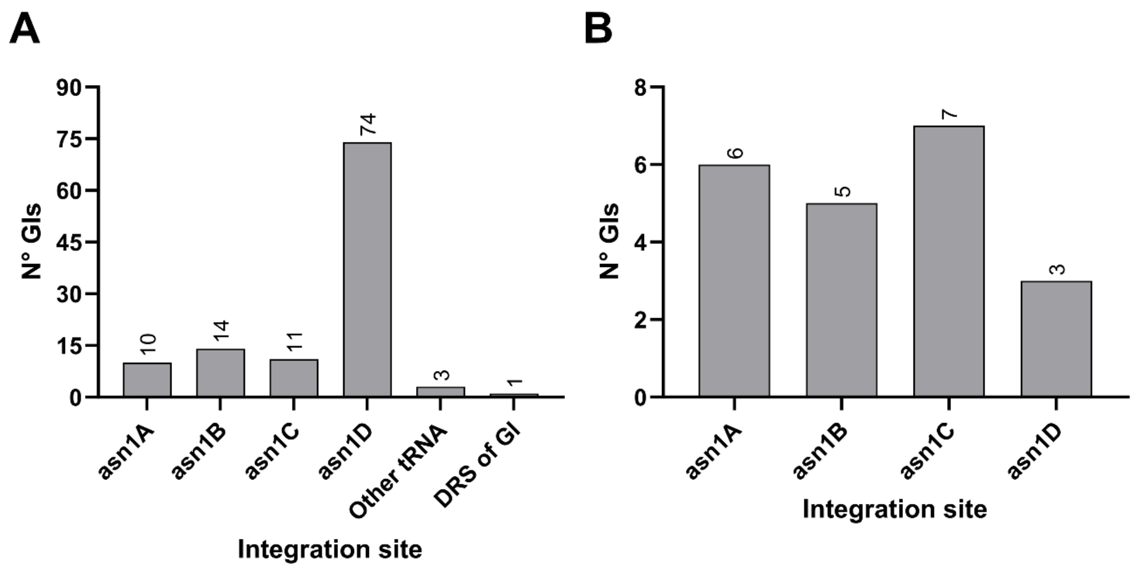

Figure S1. Integration sites used by ICEKp258.2 and ICEKp258.2-like GIs. A) Number of identified GIs and their integration sites among 113 genomes. DRS: direct-repeated sequence. **B**) Number of non-redundant GIs (the same used for the phylogeny) and their integration sites. As the *asn1A-1D* nomenclature is applied to the *K. pneumoniae* complex and *R. ornithinolytica*, the figure includes genomes from those species only. However, the GIs found in other genera were also found in tRNA-encoding genes.

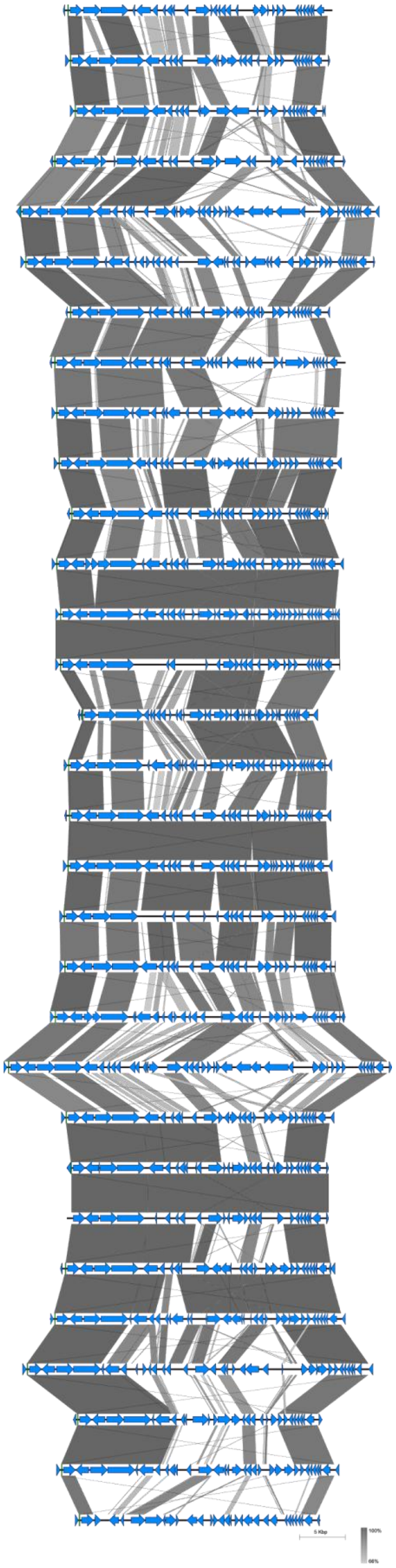

Figure S2. Sequence comparison of ICEKp258.2 and the closely related GIs identified in this study. BLASTn was performed through EasyFig v2.2.2 and the figure was drawn. This is a higher resolution version of the panel in Figure 3A. The gray lines connect the islands regions with nucleotide identity ≥66%. The color intensity corresponds to the scale.

Nucleotide identity

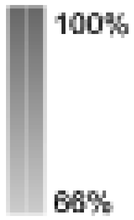

A

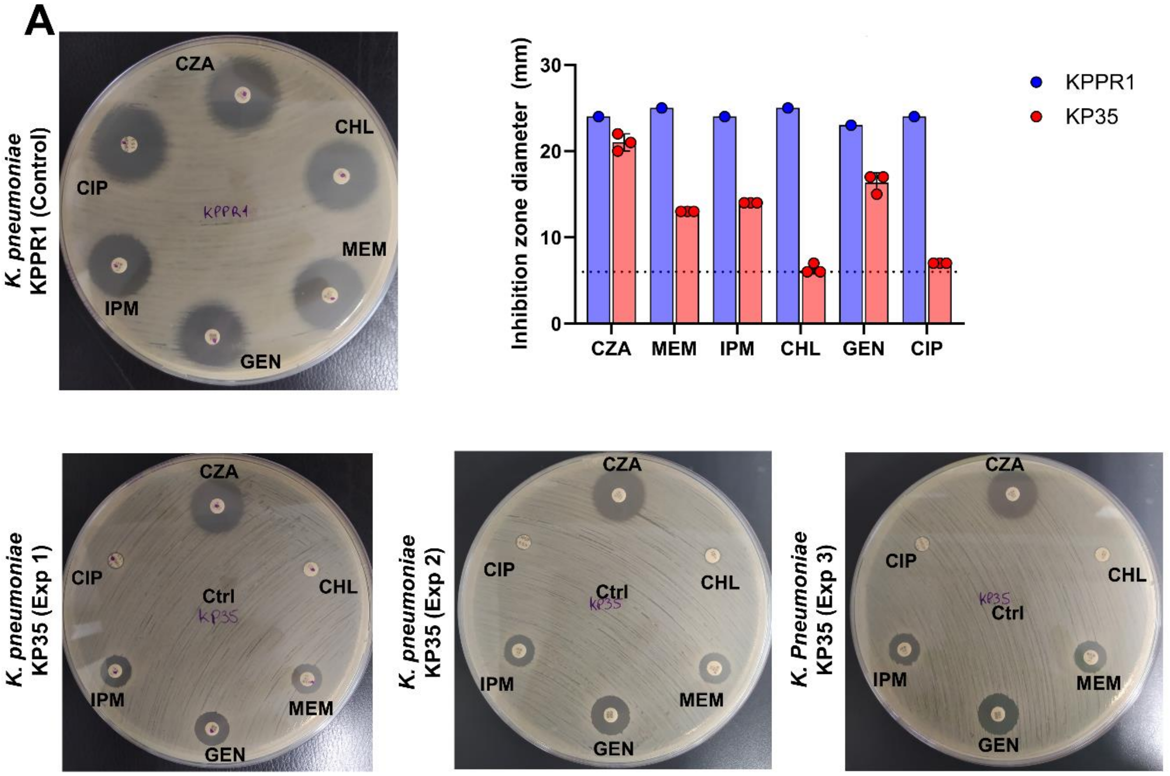

B

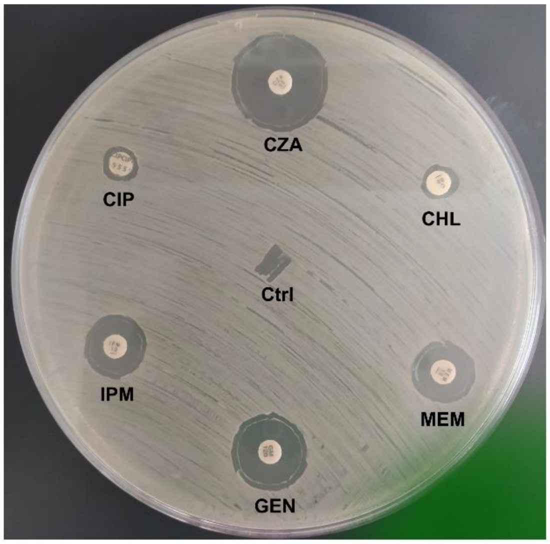

***K. pneumoniae* KP35 (Exp 2)**

After scraping 1-2 mm of the growth in the subinhibitory region

Figure S3. Challenge of *K. pneumoniae* strain KP35 with different subinhibitory antibiotic concentrations. A) KP35 was inoculated on Müeller-Hinton II agar plates (14 cm diameter), antibiotic disks were placed, and plates were incubated at 37°C during 18-20h. Three independent experiments were performed. Strain KPPR1 was used as a susceptible

control. **B)** After incubation**,** 1-2 mm of the bacterial growth occurring at the limit of the inhibition zone (or next to the disk if there was no inhibition) was scraped and used for DNA and RNA extraction. The growth at the center of the plate was scraped and used as a no-antibiotic control. Ctrl: zone used for the no-antibiotic control, CZA: ceftazidime/avibactam, MEM: meropenem, IPM: imipenem, CHL: chloramphenicol, GEN: gentamycin, CIP: ciprofloxacin.

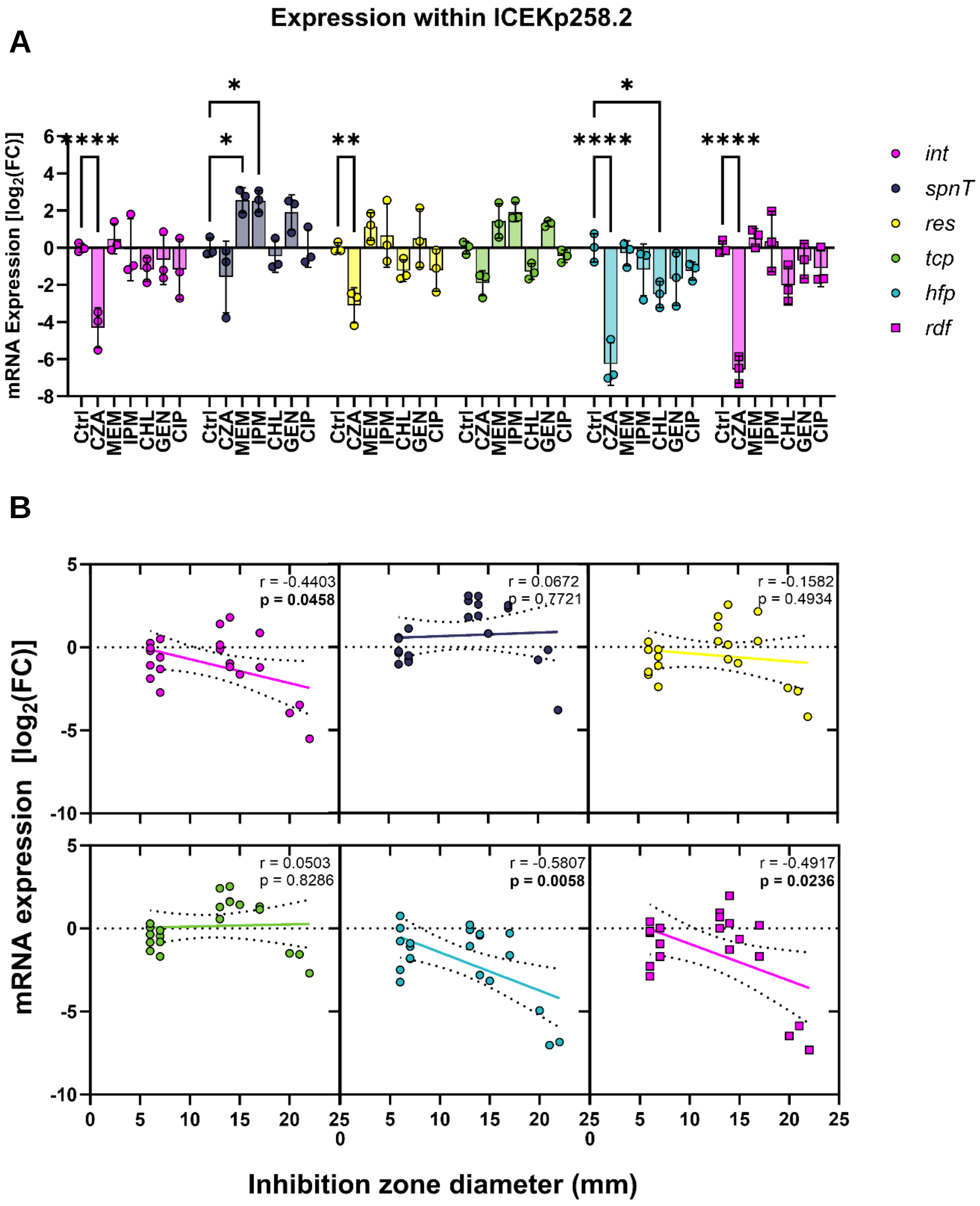

Figure S4. Expression of selected ICEKp258.2 genes in the presence of subinhibitory antibiotic concentrations. A) Expression level relative to Ctrl (non-antibiotic) treatment. This figure shows the individual data points of three independent experiments, represented as a heatmap in Fig. 5E. **B)** Analysis of the correlation between the expression and the antibiotic potency expressed as the diameter if the inhibition zone. *int*: integrase, *spnT*: SpnT, *res*: restriction endonuclease subunit of the type-III R-M system, *tcp*: TIR-domain-containing protein, *hfp*: H-NS homolog Hfp, *rdf*: recombination directionality factor. Statistical

significance in **A** was assessed through two-way ANOVA followed by Dunnett's multiple comparisons test and was indicated only when the p-value was >0.05 (*p<0.05, **p<0.01, ***p<0.001, ****p<0.0001). Pearson correlation and linear regression were used in **B**.

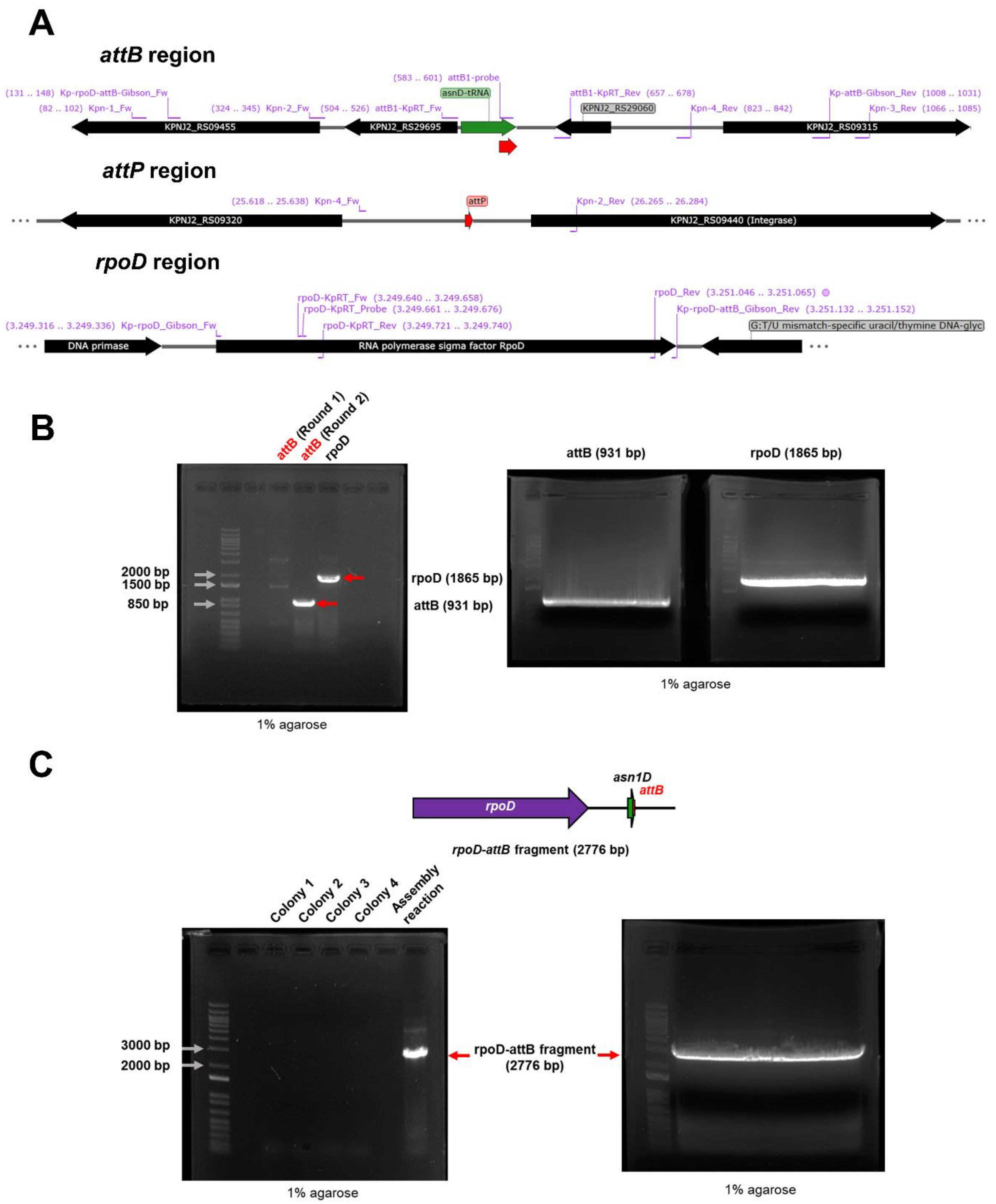

Figure S5. Location of primers and amplification of the *attB* and *rpoD* DNA fragments for construction of the *rpoD-attB* fragment. A) Location of the primers in the *attB* and *rpoD* regions. **B)** Agarose gel showing the amplification products of the *attB* region and *rpoD* gene that were joined by Gibson assembly. **C)** Agarose gel showing the amplification of the assembly product (Assembly reaction lane) that was then purified and serially diluted from 10^8 to 10^0 copies/µL and used as the standard curve for quantification of the excision of ICEKp258.2.
